# NS-187 (INNO-406), a Bcr-Abl/Lyn Dual Tyrosine Kinase Inhibitor

**Published:** 2007-11-14

**Authors:** Tomoko Niwa, Tetsuo Asaki, Shinya Kimura

**Affiliations:** 1 Discovery Research Laboratories, Nippon Shinyaku Co., Ltd. 14, Nishinosho-Monguchi-Cho, Kisshoin, Minami-ku, Kyoto 601-8550, Japan.; 2 Department of Transfusion Medicine and Cell Therapy, Kyoto University Hospital, 54 Kawahara-cho, Shogoin, Sakyo-ku, Kyoto 600-8507, Japan.

**Keywords:** NS-187, INNO-406, imatinib, chronic myeloid leukemia, Bcr-Abl, Lyn

## Abstract

Protein kinases catalyze the transfer of the γ-phosphoryl group of adenosine triphosphate (ATP) to the hydroxyl groups of protein side chains, and they play critical roles in regulating cellular signal transduction and other biochemical processes. They are attractive targets for today’s drug discovery and development, and many pharmaceutical companies are intensively developing various kinds of protein kinase inhibitors. A good example is the recent success with the Bcr-Abl tyrosine kinase inhibitor imatinib mesylate (Gleevec^™^) in the treatment of chronic myeloid leukemia. Though imatinib has dramatically improved the treatment of Bcr-Abl-positive chronic myeloid leukemia, resistance is often found in patients with advanced-stage disease. Several mechanisms have been proposed to explain this resistance, including point mutations within the Abl kinase domain, amplification of the *bcr-abl* gene, overexpression of the corresponding mRNA, increased drug efflux mediated by P-glycoprotein, and activation of the Src-family kinase (SFK) Lyn. We set out to develop a novel drug whose affinity for Abl is higher than that of imatinib and whose specificity in inhibiting Lyn is higher than that of SFK/Abl inhibitors such as dasatinib (Sprycel^™^) or bosutinib (SKI-606). Our work has led to the development of NS-187 (INNO-406), a novel Abl/Lyn dual tyrosine kinase inhibitor with clinical prospects. To provide an overview of how a selective kinase inhibitor has been developed, this review presents chemical-modification studies carried out with the guidance of molecular modeling, the structural basis for the high potency and selectivity of NS-187 based on the X-ray structure of the NS-187/Abl complex, and the biological profiling of NS-187, including site-directed mutagenesis experiments.

## Introduction

**Figure 1:**
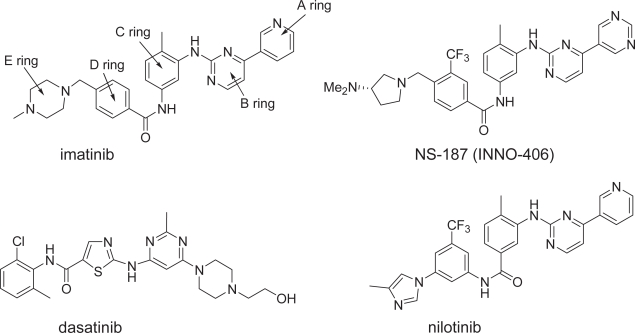
Chemical structures of Abl kinase inhibitors.

Protein kinases play critical roles in regulating cellular signal transduction and other biochemical processes by catalyzing the transfer of the γ-phosphoryl group of adenosine triphosphate (ATP) to the hydroxyl groups of protein side chains. They are therefore attractive targets for today’s drug discovery and development, and many pharmaceutical companies are intensively developing kinase inhibitors that may have therapeutic value (Cohen, 2002). A good example is imatinib mesylate (Gleevec^™^), a specific inhibitor of breakpoint cluster region—Abelson tyrosine kinase (Bcr-Abl TK) (Buchdunger et al. 1996). Imatinib (Fig. 1) is efficacious in the treatment of Philadelphia-chromosome—positive (Ph^+^) leukemias such as chronic myeloid leukemia and Ph^+^ acute lymphoblastic leukemia (Goldman et al. 2003; Kimura et al. 2006). Philadelphia chromosome is a specific chromosomal abnormality resulting from a reciprocal translocation between chromosomes 9 and 22. This translocation fuses the *c-abl* proto-oncogene to *bcr*, leading to the production of a Bcr-Abl fusion protein that constitutively activates multiple signaling pathways. Because most patients with chronic myeloid leukemia have this abnormality, Bcr-Abl tyrosine kinase is a promising target for treating Ph^+^ leukemias (Sawyers, 1999).

Within a few years of its introduction to the clinic, imatinib had dramatically altered the first-line therapy for chronic myeloid leukemia, because most patients newly diagnosed with this disease in the chronic phase achieve durable responses when treated with imatinib (O’Brien et al. 2003). However, a small percentage of these patients, as well as most patients with advanced-phase chronic myeloid leukemia and Ph^+^ acute lymphoblastic leukemia, relapse on imatinib therapy (Druker et al. 2002; Ottmann et al. 2002). Several mechanisms have been proposed to explain the cases of refractory disease and relapse, including point mutations within the Abl kinase domain, amplification of the *bcr-abl* gene, overexpression of the corresponding mRNA (Gorre et al. 2001; Hofmann et al. 2002; Nardi et al. 2004; Deininger et al. 2005), increased drug efflux from the target cells mediated by P-glycoprotein (P-gp) (Hegedus et al. 2002), and activation of Lyn, a Src-family protein kinase (SFK) (Donato et al. 2003; Dai et al. 2004; Ptasznik et al. 2004).

To overcome imatinib resistance, higher doses of imatinib and combination therapy with other agents have been used, with some efficacy. However, these strategies are limited in their application and effectiveness, especially for patients with mutations in the Abl kinase domain (Cortes et al. 2003; Kantarjian et al. 2004; O’Brien et al. 2003b). Therefore it is necessary to develop more-effective Abl TK inhibitors. Several SFK inhibitors from various chemical classes, including PD166326 (Wisniewski et al. 2002), SKI-606 (Golas et al. 2003), AP23464 (O’Hare et al. 2004), and dasatinib (Sprycel^™^; formerly BMS-354825) (Shah et al. 2004) have been reported to be 100–300 times more effective than imatinib in blocking Bcr-Abl TK autophosphorylation, and this inhibition of autophosphorylation extends to point mutants of Bcr-Abl. However, while imatinib binds only to the inactive form of Bcr-Abl, these SFK/Abl inhibitors bind also to the active form, which shares considerable conformational similarity with the active forms of diverse kinases, including the SFKs (Nagar et al. 2003). This characteristic of SFK/Abl inhibitors has some advantage with respect to Lyn kinase, because overexpression of Lyn may be associated with imatinib resistance (Donato et al. 2003; Dai et al. 2004; Ptasznik et al. 2004). However, the effects of lower specificity against SFKs are not yet fully understood, because these kinases play many important roles *in vivo* (Cary et al. 2002; Davis et al. 2003; Tanaka et al. 1996; Touyz et al. 2001). In addition to these SFK/Abl inhibitors, nilotinib (Tasigna^™^; formerly AMN107) has been developed as a novel Abl TK inhibitor. The *in vitro* inhibitory effect of nilotinib is 10–30 times greater than that of imatinib, but it is weaker than that of SFK/Abl inhibitors (Weisberg et al. 2005). Therefore, we set out to develop a drug whose affinity for Abl is higher than that of imatinib and whose specificity in inhibiting Lyn at clinically relevant concentrations without affecting the phosphorylation of other SFKs is greater than that of other SFK/Abl inhibitors.

## Structural Analysis of Kinases

Protein kinases are attractive targets for drug discovery programs in many disease areas, and most kinase inhibitors under development act by directly competing with ATP at the ATP-binding site of kinases. However, there are more than 500 protein kinases (Manning et al. 2002), and the ATP-binding site is highly conserved among them. Selectivity is therefore an essential requirement for clinically effective drugs targeted against protein kinases, and it is crucial to understand the structural characteristics of the ATP-binding site. Because kinase inhibitors on the market and currently under development often lack a portion to interact with the phosphate-binding region of the ATP-binding site, the term “ligand-binding site” will be used hereinafter instead of the term “ATP-binding site”.

**Figure 2:**
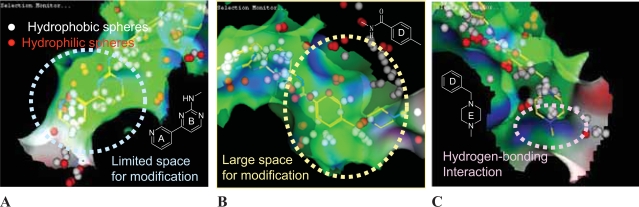
Structural properties of Abl kinase ligand-binding sites. Spheres indicate the probable locations of ligand atoms, and are classified as either hydrophilic (red) or hydrophobic (white) depending on whether or not they are in good hydrogen-bonding locations. The surface colors green, blue, and red represent the hydrophobic, hydrophilic, and exposed nature of the surface, respectively.

X-ray crystallography is a promising method for understanding the structural and physicochemical characteristics of the ligand-binding sites of protein kinases, and we have closely examined the X-ray structure of the imatinib/Abl complex. We first explored the ligand-binding site by using the 3D atomic coordinates of Abl kinase (Nagar et al. 2002), and then we calculated the surface properties of the binding site with a molecular modeling suite of MOE (Chemical Computing Group, Inc.). In Figure 2, the spheres indicate the predicted locations of an inhibitor’s atoms, and imatinib is shown for reference. The spheres are classified as either hydrophilic (red) or hydrophobic (white) depending on whether or not they are in good hydrogen-bonding locations. Green, blue, and red express the hydrophobic, hydrophilic, and exposed nature of the surface, respectively. A large part of the surface is shown in green, indicating the hydrophobic nature of the ligand-binding site of Abl.

The five rings in imatinib were labeled A through E as shown in Figure 1, and the region of the binding site around the A and B rings is shown in Figure 2A. The positions and properties of the spheres in this region correspond well to those of the A and B rings of imatinib, and there is limited space for chemical modification. There is also limited space around the C ring. In contrast, much space is available around the D ring, suggesting the feasibility of an intensive program of chemical modification of this part of the molecule (Fig. 2B). The surface color of the part of the binding site adjacent to the terminal E ring of imatinib is blue, indicating that hydrogen-bonding interactions are probably important for the binding of the E ring (Fig. 2C).

Though X-ray crystallography is very helpful for understanding the structural and physicochemical characteristics of ligand-binding sites, a sufficient number of X-ray crystallographic structures for exhaustive comparison of the binding sites of various kinases is not yet available. The only data generally available for any kinase is the amino acid sequence, and, accordingly, sequence similarity is widely used for classifying proteins and predicting biological activities (Hank et al. 1988; Hank et al. 1991). However, it is difficult to elucidate the structural characteristics of a ligand-binding site from the amino acid sequence alone.

**Figure 3:**
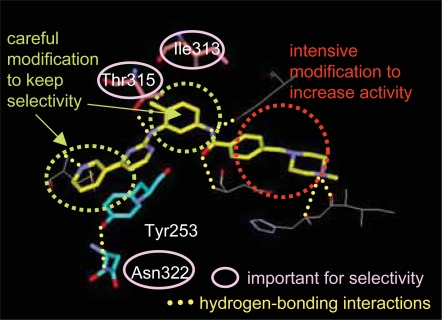
Guidelines for chemical modification.

We have developed a procedure to overcome this difficulty by using physicochemical descriptors of amino acids in conjunction with neural network modeling (Niwa, 2006). The physicochemical properties of amino acids were expressed by hydrophobic, steric and structural descriptors. Kinases are classified into four major groups based on sequence similarity, AGC (PKA, PKG, and PKC families), CaMK (calcium/calmodulin-dependent protein kinases), CMGC (CDK, MAPK, GSK3, and CLK families), and TK (Hank et al. 1988). Abl kinase belongs to the TK group, so we aimed to elucidate which amino acids and which properties characterize the ligand-binding sites of TK, and to visualize the results by molecular graphics. TK ligand-binding sites are characterized by the branched nature of the side chains of the amino acids at positions 313 and 315. The methyl group of the C ring of imatinib and similar tyrosine kinase inhibitors, known as the “flag methyl”, makes a large contribution to both their inhibitory activity and their selectivity (Zimmermann et al. 1997). The C ring and the flag methyl are located close to the amino acids at positions 313 and 315. Another characteristic feature of the TK ligand-binding site is the short side chain of the amino acid at position 322, which forms a hydrogen bond with Tyr253 and helps to stabilize the inactive conformation of Bcr-Abl kinase. Based on the above results, we developed guidelines for the chemical modification of Abl kinase ligands (Fig. 3). Though they are rather rough guidelines, they have helped us to understand the structural characteristics of the binding site of Abl kinase. In addition, they helped us to decide at the beginning of the project which kinds of chemical modifications were likely to be useful.

## Chemical Modification

To guide our chemical-modification studies, we used the reported X-ray structure of the imatinib/Abl complex (Nagar et al. 2002). When we closely examined the structure, we found a hydrophobic pocket formed by amino acids Ile293, Leu298, Leu354 and Val379 around the phenyl (D) ring of imatinib. To improve the antiproliferative activity of imatinib against Bcr-Abl-positive (Bcr-Abl^+^) leukemia cell lines, we focused on this hydrophobic pocket and introduced various hydrophobic substituents on the phenyl (D) ring (Asaki et al. 2006). We found that 3-halogenated and 3-trifluoromethylated derivatives have significantly increased inhibitory activity compared to unsubstituted imatinib (Table 1).

To compensate for the increase in hydrophobicity caused by the introduction of the hydrophobic trifluoromethyl group in **5e**, the distal pyridine (A) ring was selected for further modification. In the crystal structure of the imatinib/Abl complex, Tyr253 is located very close to the A ring, and their interaction helps to stabilize the inactive form of the kinase. Therefore, a structural modification that increases the bulk in this region would be expected to be unfavorable. The pyridine ring was therefore replaced by the more hydrophilic pyrimidine ring. Pyrimidine derivative **9a** displayed activity (IC_50_ = 4 nM) similar to the original pyridine derivative **5e**, showing that pyrimidinyl substitution is compatible with the retention of inhibitory activity.

**Figure 4:**
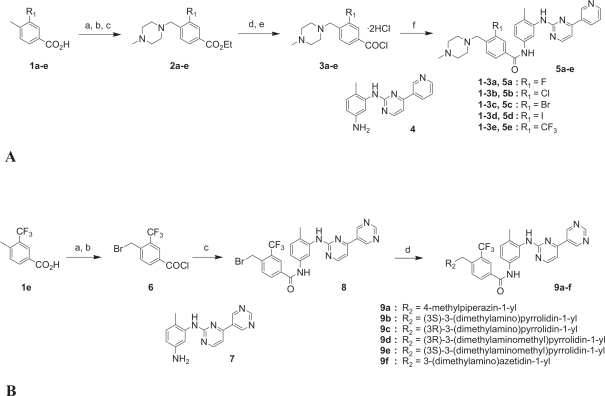
Chemical synthesis of 3-substituted benzamides. (**A**) Reagents and conditions for synthesis of **5a–e**: (a) H_2_SO_4_, EtOH, reflux; (b) NBS, cat. (PhCO)_2_O_2_, CCl_4_, reflux; (c) 1-methylpiperazine, K_2_CO_3_, THF, rt; (d) 1 N NaOH, reflux, then aq. HCl; (e) SOCl_2_, reflux; (f) **4**, pyridine, rt. (**B**) Reagents and conditions for synthesis of **9a–f**: (a) NaBrO_3_, NaHSO_3_, EtOAc; (b) (COCl)_2_, cat. DMF, CH_2_Cl_2_, rt; (c) 7, K_2_CO_3_, dioxane, rt; (d) cyclic amines, K_2_CO_3_, DMF, rt. Adapted from Asaki et al. 2006.

The crystal structure of the complex reveals that the piperazine moiety (E) of imatinib interacts with the carbonyl oxygen atoms of Ile360 and His361 through hydrogen bonding. Taking account of this important interaction, we replaced the piperazine moiety in **9a** with other cyclic amines. The optically pure 3-(dimethylamino) pyrrolidine derivatives **9b** and **9c**, the 3-(dimethylaminomethyl)pyrrolidine derivatives **9d** and **9e**, and the 3-(dimethylamino)azetidine derivative **9f**, all of whose E-ring systems had the potential to function as piperazine isosteres, were synthesized (Fig. 4). The pyrrolidine derivative **9c** exhibited excellent potency (IC_50_ = 4 nM), comparable to **9a**. Compound **9b**, the enantiomer of **9c**, and other pyridine derivatives (**9d** and **9e**) had lower, though still excellent, antiproliferative activity (IC_50_ = 11, 11 and 9 nM, respectively). Azetidine **9f** had lower potency still (IC_50_ = 17 nM). These results reveal that the antiproliferative activity is somewhat affected by the position of the terminal dimethylamino function. In other words, the differences in activity among these compounds may be attributed to subtle differences in the distance between the dimethylamino function and the carbonyl oxygen atoms of Ile360 and His361.

At the beginning of our project, the involvement of Lyn kinase in imatinib resistance was unknown. In 2003, Donato et al. reported the association of the overexpression of Lyn kinase with imatinib resistance (Donato et al. 2003; Dai et al. 2004; Ptasznik et al. 2004). Thereafter, we tried to develop Abl/Lyn dual inhibitors, and found that NS-187 (**9b**; INNO-406) and its derivatives also inhibit Lyn kinase. To investigate why this series of compounds act as dual Bcr-Abl/Lyn kinase inhibitors, we determined their inhibitory activities against Abl and Lyn kinases and studied their structure-activity relationships (Horio et al. 2007). All compounds tested show more-potent inhibitory activity against Abl and Lyn than does imatinib (Table 1), and the inhibitory activities of 3-substituted benzamides against Abl and Lyn are highly correlated (r = 0.982 when the activity is expressed as pIC_50_).

Judging from its overall characteristics, including its pharmacokinetics and toxicity as determined in animal studies, we selected **9b** (NS-187) as a candidate for clinical development (Kimura et al. 2005; Asaki et al. 2006). NS-187 is now under investigation in a Phase I clinical trial with Ph^+^ leukemia.

## Structural Analysis of NS-187

### X-ray structure of NS-187 bound to human Abl

**Figure 5:**
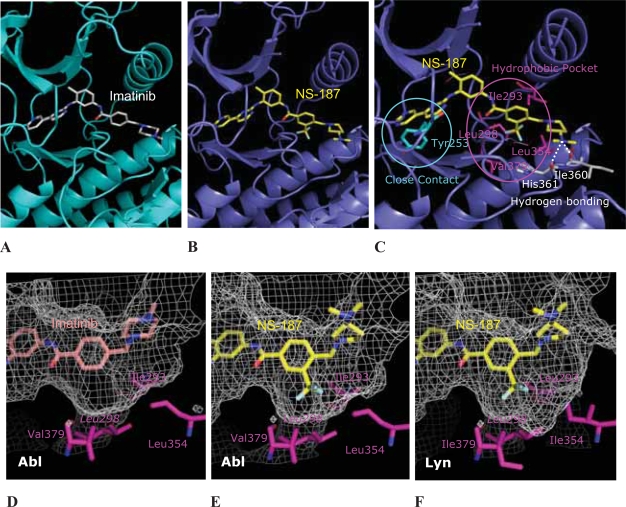
NS-187 and imatinib bound to Abl and Lyn. (**A**) and (**B**) are X-ray structures of imatinib/Abl and NS-187/Abl complexes, respectively. (**C**) shows important interactions between NS-187 and Abl. Comparison of the CF_3_ binding pocket in Abl and Lyn is shown in D–F. Meshes show the molecular surfaces of the kinases. The hydrophobic amino acids forming the hydrophobic pocket are shown in magenta. (**D**), (**E**) and (**F**) show the imatinib/Abl, NS-187/Abl, and NS-187/Lyn complexes, respectively. Adapted from Asaki et al. 2006 and Horio et al. 2007.

We recently determined the X-ray structure of NS-187 bound to human Abl (Horio et al. 2007) shown in Figure 5B (Abl, blue; NS-187, yellow). For comparison, the X-ray structure of imatinib bound to Abl (Abl, cyan; imatinib, white) is shown in Figure 5A. Only the amino acids within 4 Å of NS-187 or imatinib are depicted for clarity. The two X-ray structures resemble each other very closely, with only slight differences in the positions of the ligands and the side chains and backbones of the kinases. Therefore it is clear that NS-187 and imatinib interact with Abl in very similar ways. This finding validates our use of the X-ray structure of the imatinib/Abl complex to guide our chemical-modification studies.

We checked whether our strategy for chemical modification was appropriate by analyzing the X-ray structure of the NS-187/Abl complex (Fig. 5C). The trifluoromethyl (CF_3_) group is well placed to interact with the hydrophobic pocket formed by Ile293, Leu298, Leu354, and Val379, shown in magenta. Tyr253 is located close to the pyrimidine (A) ring, so that our use of a pyrimidine instead of a pyridine ring does not appear to alter the important role of Tyr253 in stabilizing the inactive form of the kinase. Hydrogen-bonding interactions are shown as broken white lines in Figure 5C, and it can be seen that the nitrogen atom of the dimethylamino group is well placed to interact with the carbonyl oxygen atoms of Ile360 and His361 through hydrogen bonding. Our strategy for chemical modification was thus validated.

### Effects of the CF_3_ group of NS-187

The 3-substituents (R^1^) on the D ring greatly enhance the inhibitory activity against both Abl and Lyn kinases (Table 1). To elucidate this effect, we quantitatively analyzed the effect of the 3-substituent on the inhibitory activity of the compounds against Abl and Lyn kinases by using various physicochemical parameters of the 3-substituents. We found that the inhibitory activity is highly correlated with the hydrophobic substituent parameter π (correlation coefficient r = 0.958 for Abl and r = 0.977 for Lyn) (Horio et al. 2007). This means that the inhibitory effect increases with the hydrophobicity of the 3-substituent.

To understand this effect more clearly, we examined the molecular surfaces of Abl and Lyn kinases near the 3-substituent (Fig. 5D–F). It is apparent that there remains room to accommodate chemical modification of the D ring in the hydrophobic pocket formed by the amino acids Ile293, Leu298, Leu354 and Val379, shown in magenta in the X-ray structure of the imatinib/Abl complex (Fig. 5D). However, in the X-ray structure of the NS-187/Abl complex, the CF_3_ group occupies this hydrophobic pocket well (Fig. 5E). The modeled structure of the NS-187/Lyn complex, which is based on the X-ray structure of the NS-187/Abl complex, is depicted in Figure 5F. Close to the 3-substituent there are four hydrophobic amino acids, Leu293, Leu298, Ile354 and Ile379, shown in magenta (Horio et al. 2007). Although the identities of three of the four amino acids differ between Abl and Lyn, they are all hydrophobic amino acids. Therefore it is likely that the enhanced inhibitory activity of the modified compounds against both Abl and Lyn can be explained by increased hydrophobic interactions. It is reasonable that the hydrophobic effect of the 3-substituent, as expressed by π, significantly enhances the inhibitory activity.

**Figure 6:**
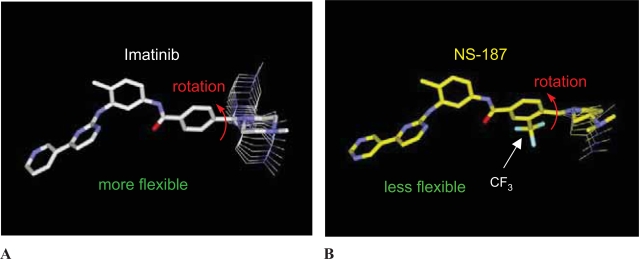
Restricted flexibility of NS-187 is shown by the rotational barrier about the bond connecting the D ring of imatinib or NS-187 and the terminal E ring. Conformers within 4 kcal/mol above the global minimum are depicted, and the stick models represent the active structures of imatinib (**A**) and NS-187 (**B**) bound to Abl kinase. Adapted from Kimura et al. 2006b.

The inhibitory activities of the compounds are also linearly correlated with the Sterimol parameter B1, which expresses the minimum width of the 3-substituent (r = 0.988 for Abl and r = 0.991 for Lyn) (Horio et al. 2007); that is, the inhibitory effect increases with the size of the 3-substituent. Since the 3-substituent is located adjacent to the terminal dimethylaminopyrrolidine ring (E), it would be expected to hinder the rotation of the terminal ring. When we calculated the rotational barrier of the terminal ring by using the MMFF94x force field with MOE, we indeed found a restricted rotation about the bond connecting the D and E rings of NS-187 (Fig. 6). The CF_3_ group not only reduces the flexibility of rotation of the terminal ring, but also helps NS-187 to adopt its bound (active) conformation (Kimura et al. 2006b). Because of the reduced loss of entropy upon binding, the inhibitory activity of the ligand would be expected to increase as the probability that it will adopt its active conformation increases. In addition, the reduced conformational flexibility could reduce the probability of binding to other proteins, thereby reducing the probability of adverse side effects.

Though kinase inhibitors bearing a CF_3_ group are not rare, those with a CF_3_ group adjacent to another group are rare. The adjacent location of these groups is a very characteristic structural feature of NS-187. The increased hydrophobicity and reduced conformational flexibility of NS-187 relative to imatinib cooperate to enhance its inhibitory activity against both Abl and Lyn kinase and reduce the probability of binding to off-target proteins.

## *In Vitro* Biological Activity of NS-187

### NS-187 blocks wild-type Bcr-Abl signaling

We compared the ability of NS-187 and imatinib to inhibit the phosphorylation of Bcr-Abl and other tyrosine kinases at the cellular level (Kimura et al. 2005). The IC_50_ values of NS-187 against wild-type Bcr-Abl in human erythroleukemia K562 cells and human embryonic kidney 293T cells are 11 and 22 nM, respectively, while the corresponding values for imatinib are 280 and 1200 nM. NS-187 is therefore 25 to 55 times more potent than imatinib in blocking Bcr-Abl autophosphorylation. NS-187 suppresses the phosphorylation of platelet-derived growth factor receptor (PDGFR) and c-Kit with a potency similar to that of imatinib. However, while the potency ranking for imatinib is PDGFR > c-Kit > Bcr-Abl, the potency ranking for NS-187 is Bcr-Abl > PDGFR > c-Kit, so that the specificity of NS-187 for Bcr-Abl is greater than that of imatinib. Because inhibition of PDGFR or c-Kit could cause unpredictable adverse effects, specific inhibition of Bcr-Abl is desirable. Examination of the intracellular phosphorylation status of CrkL and ERK, downstream mediators of the effects of Bcr-Abl, revealed that NS-187 inhibits the phosphorylation of these proteins in K562 cells at much lower concentrations than does imatinib. This inhibition of phosphorylation is also observed in the mouse ProB cell line BaF3 expressing wild-type Bcr-Abl (BaF3/wt). Taken together, these findings indicate that NS-187 is much more potent and specific than imatinib in blocking the effects of Bcr-Abl.

### Antiproliferative activity of NS-187 against cells bearing wild-type or mutated Bcr-Abl

More than 40 point mutations within the Abl kinase domain have been reported (Hochhaus and Rosee, 2004). NS-187 at physiologically obtainable concentrations inhibits the phosphorylation of Bcr-Abl bearing the M244V, G250E, Q252H, Y253F, E255K, E255V, F317L, M351T, E355G, F359V, H396P, or F486S mutations, but it does not inhibit the phosphorylation of the T315I mutant (Kimura et al. 2005). Against all mutants except T315I, NS-187 is at least five times as potent as imatinib (Table 2).

NS-187 suppresses the growth of the Bcr-Abl^+^ cell lines K562, KU812 and BaF3/wt much more potently than does imatinib, but neither drug affects the proliferation of the Bcr-Abl-negative cell line U937 (Kimura et al. 2005). NS-187 exhibits a concentration-dependent antiproliferative effect against BaF3 cell lines expressing the Bcr-Abl mutants M244V, G250E, Q252H, Y253F, E255K, M351T or H396P, but has no effect on BaF3 cells expressing the T315I mutant. Bcr-Abl/wt, Q252H and M351T are especially sensitive to NS-187. Imatinib, meanwhile, is much less active against all cell lines tested (Naito et al. 2006). NS-187 therefore potently inhibits both the intracellular phosphorylation of most mutated Bcr-Abl kinases and the proliferation of cells expressing these kinases.

### Mechanisms of NS-187-mediated cell death in Bcr-Abl^+^ leukemic cells

NS-187 augments the activity of pro-apoptotic Bcl-2 homology domain 3 (BH3)-only proteins and induces apoptosis in Bcr-Abl^+^ leukemic cells, as evidenced by DNA fragmentation, caspase-3 activation, and the loss of mitochondrial-outer-membrane permeabilization (Kuroda et al. 2007). ABT-737, an inhibitor of Bcl-2 and Bcl-XL, enhances the apoptosis induced by NS-187, even in cells with mutated Bcr-Abl that are less sensitive to NS-187, suggesting that Bcl-2-family-regulated, intrinsic apoptosis occurs through caspase activation. Even in the presence of the pan-caspase inhibitor zVAD-fmk, NS-187 still induces apoptosis in some cells, indicating the additional involvement of NS-187 in a caspase-independent apoptotic pathway. The observation of an increased number of cells showing the hallmarks of autophagy suggests that autophagy participates in the response against Bcr-Abl blockade. Inhibition of autophagy by chloroquine significantly enhances NS-187-induced cell death. These results may be useful in the design of a rational therapeutic approach for efficiently eradicating Bcr-Abl^+^ leukemic cells.

### Inhibition of phosphorylated Abl by NS-187

Imatinib inhibits the kinase activity of the Tyr393-unphosphorylated form of the Abl kinase domain with an IC_50_ value of 35 nM but has little effect on the phosphorylated form. In contrast, NS-187 effectively inhibits the kinase activity of both Tyr393-phosphorylated and Tyr393-unphosphorylated forms of Abl with respective IC_50_ values of 72 nM and 30 nM, suggesting that NS-187 may have sufficiently high affinity for Bcr-Abl to enable it to bind even to an unfavourable conformation of the kinase (Naito et al. 2006).

### Selectivity of NS-187 for Abl

A panel of 79 tyrosine kinases, including the five Src-family proteins Blk, Src, Fyn, Lyn and Yes, was assayed in the presence and absence of NS-187 or imatinib (Kimura et al. 2005). Concentrations of 0.1 μM NS-187 and 10 μM imatinib were used because these concentrations give equal inhibition of Abl. At 0.1 μM, NS-187 strongly inhibits only three of the 79 tyrosine kinases, that is, Abl, Arg and Lyn (Fyn is less strongly inhibited). At this concentration, NS-187 does not inhibit PDGFRα, PDGFRβ, Blk, Src or Yes. In contrast, 10 μM imatinib inhibits nine tyrosine kinases, that is, Abl, Arg, Blk, Flt3, Fyn, Lyn, PDGFRα, PDGFRβ and p70S6K. NS-187 therefore inhibits Abl more selectively than does imatinib. The IC_50_ values of NS-187 for Abl, Src and Lyn are 5.8, 1700 and 19 nM, respectively, while those of imatinib are 106, >10,000 and 352 nM, respectively. These findings suggest that NS-187 acts as an Abl/Lyn dual inhibitor while otherwise remaining highly specific.

## *In Vivo* Anti-Tumor Activity of NS-187

### Activity of NS-187 in mouse tumor models

The ability of NS-187 to suppress tumor growth was tested in two murine tumor models (Kimura et al. 2005). In one model, Balb/c-nu/nu mice were injected subcutaneously with KU812 cells on Day 0 and given NS-187 or imatinib orally twice a day from Day 7 to Day 17. At 20 mg/kg/day, imatinib inhibits tumor growth slightly, while at 200 mg/kg/day, it inhibits tumor growth almost completely. NS-187, meanwhile, significantly inhibits tumor growth at only 0.2 mg/kg/day, while at 20 mg/kg/day it completely inhibits tumor growth without adverse effects. When mice were treated with NS-187 at 0.2 or 20 mg/kg/day, the estimated C_max_ was 4 or 400 nM, respectively, comparable to the concentrations at which the *in vitro* effects of NS-187 are obtained. NS-187 is therefore at least 10-fold more potent than imatinib *in vivo* with complete inhibition of tumor growth as the end-point and at least 100-fold more potent with partial inhibition as the endpoint. NS-187 was well tolerated by the mice.

In the other model, Balb/c-nu/nu mice intravenously injected with BaF3/wt cells were given NS-187 or imatinib orally for 11 days starting on Day 1. All seven untreated mice had died by Day 23 due to leukemic cell expansion, while all mice treated with 400 mg/kg/day imatinib had died by Day 25. NS-187, in contrast, significantly prolonged the survival of the mice in a dose-dependent manner compared with untreated mice.

**Figure 7:**
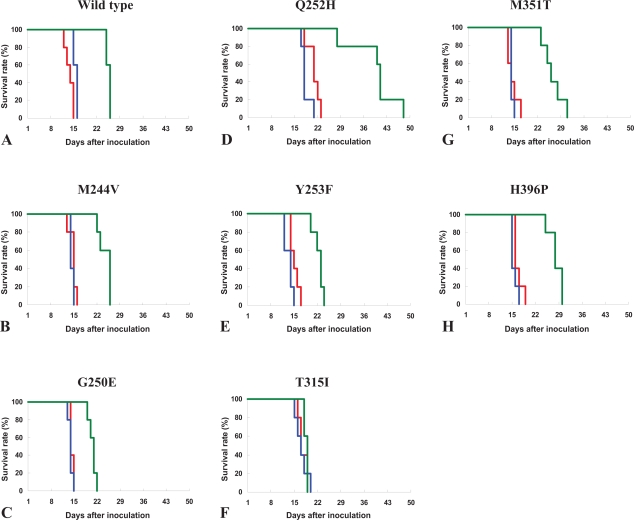
*In vivo* activity of NS-187 in an imatinib-resistant mouse leukemia model. (**A**–**H**) BALB/cA-nu mice were intravenously injected with Ba/F3 cells expressing the indicated mutant forms of Bcr-Abl on day 1. The mice were orally administered twice daily with NS-187 (green), imatinib (blue) or vehicle (red) from day 2 through day 12. The survival of the mice was assessed by the method of Kaplan and Meier. Adapted from Naito et al. 2006.

To investigate the efficacy of NS-187 in a mouse model of leukemia, we tested its ability to block the growth of BaF3 cells expressing mutated Bcr-Abl in Balb/c-nu/nu mice (Naito et al. 2006). Mice bearing BaF3 cells expressing M244V, G250E, Q252H, Y253F, E255K, T315I, M351T or H396P were treated with NS-187 or imatinib. Mice bearing BaF3 cells expressing wild-type Bcr-Abl or any mutant form of Bcr-Abl except T315I show significant prolongation of survival when they receive NS-187 at a dosage of 200 mg/kg/day, without any apparent signs of toxicity (Fig. 7). These *in vivo* results are consistent with the *in vitro* results. Imatinib, even at a dosage of 400 mg/kg/day, is much less effective. NS-187 results in the highest observed percentage increase in mean survival in mice bearing BaF3 cells expressing wild-type Bcr-Abl, Q252H or M351T, in good agreement with the *in vitro* results. More-over, the rank-order of the IC_50_ values for cell growth inhibition is inversely correlated with the percentage increase in the mean survival of mice treated with NS-187. Thus, the efficacy of NS-187 in the mouse leukemia model mirrors its *in vitro* activity, a result which suggests that NS-187 will be clinically effective.

### Activity of NS-187 against central nervous system leukemia

**Figure 8:**
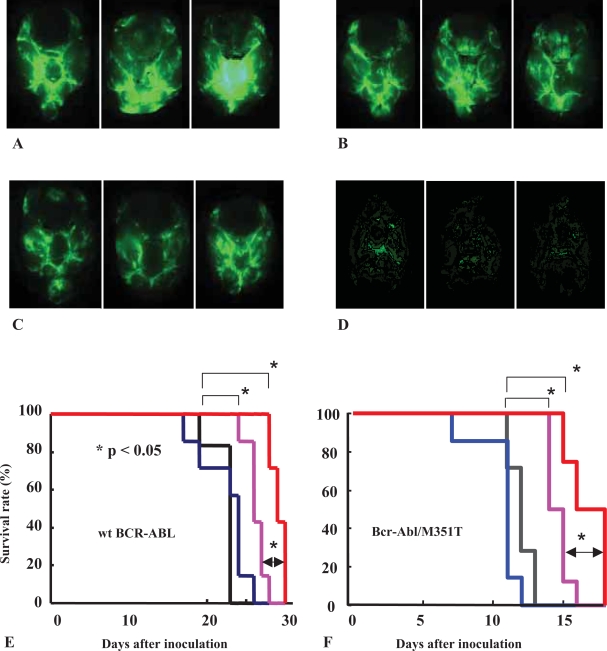
Combined effect of CsA and NS-187 in an *in vivo* CNS leukemia model. BALB/cA Jcl-*nu* mice were inoculated into the right cerebral ventricle with 5 × 10^4^ BaF3/wt *bcr-abl*^GFP^ (A–E) or BaF3/*bcr-abl*/M351T (F) cells on day 0. Mice were orally administered vehicle (A, black line in E and F), 50 mg/kg CsA (B, blue line in E and F), 60 mg/kg/day NS-187 (C, magenta line in E and F), or a combination of CsA and NS-187 (D, red line in E and F) from day 5 to day 15. Brains from each group were taken on day 17. Adapted from Yokota et al. 2007.

Because the penetration of imatinib into the central nervous system (CNS) is poor, the CNS can become a sanctuary site of relapse in patients on prolonged imatinib therapy. P-gp plays an important role in limiting the distribution of imatinib to the CNS, and it is well known that imatinib is a substrate for P-gp. Our preliminary pharmacokinetic study (Yokota et al. 2007) showed that the intracranial concentration of NS-187 is only 10% of its serum concentration, suggesting the involvement of P-gp. However, even though NS-187 is a substrate for P-gp, it still inhibits the proliferation of leukemic cells in the brain, whereas imatinib does not. NS-187 significantly prolongs the survival of mice in a dose-dependent manner in two CNS leukemia murine models compared with imatinib. Furthermore, cyclosporine A, a P-gp inhibitor, augments the *in vivo* activity of NS-187 against CNS Ph^+^ leukemia, as shown by whole-brain fluorescence imaging (Fig. 8A–D) and survival curves (Fig. 8E and F). These findings indicate that NS-187 is a promising agent for the treatment of CNS Ph^+^ leukemia.

### Phase I clinical study of NS-187 (INNO-406)

A phase I study of NS-187 (INNO-406) in 21 patients with Ph^+^ leukemia who were resistant to or intolerant of imatinib is in progress (Kantarjian et al. 2007).

## Summary and Conclusions

Using X-ray crystallographic information and computer modeling, we have developed a highly potent and selective Abl/Lyn dual tyrosine kinase inhibitor, NS-187 (INNO-406). Its characteristic structural features are a trifluoromethyl group on the D ring that occupies a hydrophobic pocket of the Abl ligand-binding site and an adjacent dimethylaminopyrrolidine E ring whose rotation is restricted by the trifluoromethyl group. These features not only enhance inhibitory activity against Abl but also increase selectivity by reducing binding to off-target proteins. NS-187 has higher potency in inhibiting Abl than does imatinib and higher selectivity in inhibiting Lyn than do other SFK/Abl inhibitors. NS-187 is less sensitive to point mutations in the Abl kinase domain than are other inhibitors such as imatinib, while maintaining a high selectivity for Abl and Lyn. NS-187 may be effective in the treatment of chronic myeloid leukemia with possible application to CNS leukemia and it may also be less liable to cause unfavorable side effects than are therapeutic agents that target multiple kinases, such as SFK inhibitors.

## Figures and Tables

**Table 1.** Inhibitory and antiproliferative activity of 3-substituted benzamide derivatives.

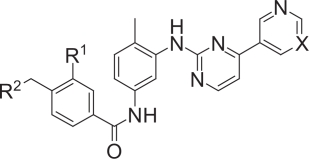							
**Compound**	**X**	**R^1^**	**R^2^**	**IC_50_ (nM)**							
				**K562 cells (Bcr-Abl)^a^**	**U937 cells^a^**	**Abl^b^**	**Lyn^b^**
imatinib	CH	H	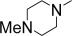	182	14,000	220	470
5a	CH	F	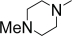	63	8,000	35	120
5b	CH	Cl	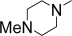	10	9,000	N.D.	N.D.
5c	CH	Br	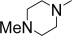	7	5,000	3.1	12
5d	CH	I	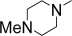	10	6,000	N.D.	N.D.
5e	CH	CF_3_	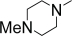	5	4,000	4.8	5.1
9a	N	CF_3_	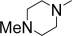	4	5,000	8.5	8.8
9b (NS-187)	N	CF_3_	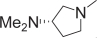	11	10,000	11	26
9c^d^	N	CF_3_	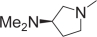	4	9,000	3.4	12
9d^d^	N	CF_3_	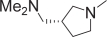	11	20,000	N.D.	N.D.
9e^d^	N	CF_3_	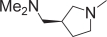	9	>100,000	7.6	32
9f^d^	N	CF_3_	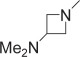	17	>100,000	29	140

a IC_50_ represents the concentration of compound which inhibits cell proliferation by 50%.

b IC_50_ represents the concentration of compound which inhibits the kinase activity by 50%.

c ND, not determined.

d The biological activity of the monohydrochloride salts was evaluated. Adapted from Asaki et al. 2006 and Horio et al. 2006.

**Table 2:** Effect of NS-187 and imatinib on *in vitro* phosphorylation of purified wild-type and point-mutated Abl kinase domains.

**Abl kinase**	**IC50 (nM)**	
	**NS-187**	**imatinib**
Wild type	72	1,100
M244V	240	3,500
G250E	160	2,000
Q252H	410	2,100
Y253F	81	1,500
E255K	540	5,800
E255V	1,400	>10,000
T315I	>10,000	>10,000
F317L	760	1,900
M351T	150	3,900
E355G	580	7,100
E359V	1,300	>10,000
H396P	95	1,400
F486S	470	9,500

Adapted from Kimura et al. 2005.
